# Longitudinal Relationships of Religion with Posttreatment Depression Severity in Older Psychiatric Patients: Evidence of Direct and Indirect Effects

**DOI:** 10.1155/2012/745970

**Published:** 2012-02-22

**Authors:** R. David Hayward, Amy D. Owen, Harold G. Koenig, David C. Steffens, Martha E. Payne

**Affiliations:** ^1^Department of Health Behavior and Health Education, School of Public Health, University of Michigan, Ann Arbor, MI 48109-2029, USA; ^2^Center for Spirituality, Theology and Health, Duke University Medical Center, Durham, NC, USA; ^3^Department of Psychiatry and Behavioral Sciences, Duke University Medical Center, Durham, NC, USA; ^4^Department of Medicine, Duke University Medical Center, Durham, NC, USA; ^5^Neuropsychiatric Imaging Research Laboratory, Duke University Medical Center, Durham, NC, USA

## Abstract

Psychiatric patients (age 59+) were assessed before study treatment for major depressive disorder, and again after 3 months. Measures taken before study treatment included facets of religiousness (subjective religiosity, private prayer, worship attendance, and religious media use), social support, and perceived stress. Clinician-rated depression severity was assessed both before and after treatment using the Montgomery-Åsberg Depression Rating Scale (MADRS). Structural equation modeling was used to test a path model of direct and indirect effects of religious factors via psychosocial pathways. Subjective religiousness was directly related to worse initial MADRS, but indirectly related to better posttreatment MADRS via the pathway of more private prayer. Worship attendance was directly related to better initial MADRS, and indirectly related to better post-treatment MADRS via pathways of lower stress, more social support, and more private prayer. Private prayer was directly related to better post-treatment MADRS. Religious media use was related to more private prayer, but had no direct relationship with MADRS.

## 1. Introduction

A substantial body of research has linked religiousness with better mental health [1–3], particularly in older adulthood [4–6]. More intensely religious individuals have been found to be at lower risk for depression [7, 8] and, for those who do become depressed, to experience less severe symptoms [9–11] and faster remission [12, 13]. However, the nature of this relationship is complicated by a growing body of contrary findings that religion may be related to worse outcomes in some groups [8, 14], or that in some circumstances there may be a curvilinear relationship, with moderate levels of religiousness conferring the most benefits [9, 15]. Some of these discrepancies may be explained by the complexity of the construct of religion, which entails multiple aspects of belief and behavior such as participation in public worship services, engagement in private prayer, and perceiving order and meaning in one's life. While these phenomena may be highly correlated, they may also have distinct and even contrasting psychological implications. For example, one recent study of lifetime risk of major depression found that while more frequent attendance at religious services appeared to be a protective factor, reporting a strong relationship with God was associated with more risk of depression [16]. Another study similarly found service attendance linked with lower risk of depression, but stronger self-rated religiousness associated with greater risk [17]. Both were cross-sectional studies, however.

There is evidence that psychosocial processes, particularly social support and stress buffering, mediate a significant proportion of the relationship between religion and depression [18]. Participation in the activities of a congregation or other religious community can provide psychological as well as material support from other members [19, 20]. At the same time, religious beliefs can provide internal coping resources that help to reduce psychological stress, such as a sense of meaning and purpose, and ways of reframing negative life events [21]. Relatively few studies have directly assessed these theoretical pathways of mediation with respect to depression, but one found that congregational support and religious coping mediated the respective effects of service attendance and prayer on symptoms of depression in the general population [22]. Another found that social support and religious coping both played a role in mediating the impact of general religiousness on postoperative psychological distress in cardiac surgery patients [23].

**Figure 1:**
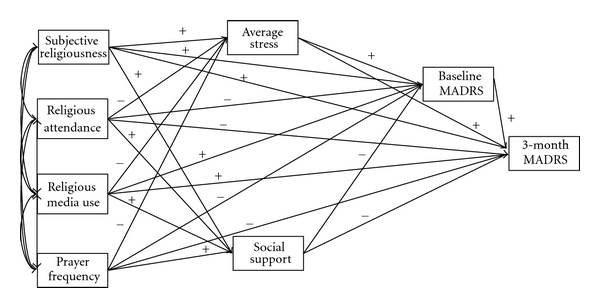
Theoretical path model for effects of religious factors on depression severity. MADRS: Montgomery-Åsberg Depression Rating Scale.

The purpose of the present study is to examine the longitudinal relationship between baseline religious factors and outcomes following treatment for depression, and the psychosocial pathways by which they may operate. The sample consists of depressed psychiatric patients, who were assessed prior to treatment and again after three months. Pretreatment religious and psychosocial characteristics are used to construct a mediational path model of posttreatment depression severity. Based on previous research, it was hypothesized that religious factors (including service attendance, subjective religiousness, prayer frequency, and use of religious media) would be related to greater social support and less stress, which would in turn be related to less severe depression after treatment (Figure 1).

## 2. Materials and Method

### 2.1. Setting and Patients

Participants were enrolled in the Neurocognitive Outcomes of Depression in the Elderly (NCODE) study between November 1994 and December 2008. Methodological details of NCODE, a prospective cohort study conducted at Duke University Medical Center, have been described previously [24]. All participants were at least 59 years of age and patients of Duke Psychiatric Services, receiving treatment for a current episode of unipolar depression, without evidence of other major psychiatric illness. On enrollment, participants were interviewed by a geriatric psychiatrist, which included administration of the Montgomery-Åsberg Depression Rating Scale (MADRS) [25] described below, and this procedure was repeated during the course of treatment at intervals of three months or less. At or shortly after the point of enrollment, participants completed all other measures used in the present study as part of the Duke Depression Evaluation Schedule (DDES) [26], administered in-person by trained interviewers. All subjects provided informed consent before beginning any study procedures, and all study procedures were approved by the Duke University Health System Institutional Review Board.

### 2.2. Measures

#### 2.2.1. Depression Severity

Severity of depression was measured using the MADRS [25], which is a clinician-rated instrument designed to be sensitive to changes resulting from treatment, and which has high interrater reliability [27]. The MADRS has a total range of 0–60, with established grades of depression severity corresponding to scores 0–6 (recovered), 7–19 (mild), 20–34 (moderate), and 35–60 (severe) [28].

#### 2.2.2. Religious Factors

Five religious factors were measured as part of the DDES: worship attendance, religious media use, private prayer, subjective religiosity, and group affiliation. Frequency of worship attendance was measured on a 6-point scale (never, once a year or less, a few times a year, a few times a month, once a week, more than once a week). Frequency of viewing/listening to religious television or radio was measured on a 6-point scale (rarely or never, once a month, a few times a month, once a week, 2-3 times a week, daily). Frequency of private religious practice—including prayer, meditation, and Bible study—was measured on a 6-point scale (rarely or never, a few times a month, once a week, two or more times a week, daily, more than once a day). Subjective religiosity was measured with a single item on a 3-point scale of personal religious importance (very important, somewhat important, not important at all).

Participants were asked their religious group preference, including specific denomination. For Christians, these responses were coded according to definitions established previously [29] into Mainline Protestant, Conservative Protestant, and Catholic categories. Because only a small number of participants reported belonging to a non-Christian religious group, these individuals were classified together as “other religion,” while those reporting no religious preference were classified as having “no religion.”

#### 2.2.3. Social Support

The Duke Social Support Index [26, 30] was administered to all participants. The present analyses use the 10-item subjective social support subscale, measuring perceptions of being included in a social network (e.g., “When you are talking with your family and friends, do you feel you are being listened to most of the time, some of the time, or hardly ever?”).

#### 2.2.4. Stress

Perceived stress were measured with a single interview item: “On a scale of 1 to 10, how would you rate the average degree of stress you have experienced during the past six months? One means no stress whatsoever and a 10 means you had stress so severe that you were unable to cope with everyday activities.”

#### 2.2.5. Vascular Health

The presence and severity of comorbid vascular health conditions was measured with a series of 4 questions in which participants were asked whether they currently had specific vascular health conditions (“high sugar or diabetes,” “heart trouble,” “high blood pressure or hypertension,” and “hardening of the arteries”). For each vascular condition reported, participants were asked whether it interfered with their activities “not at all,” “a little,” or “a lot.” Responses to these items were added to create a combined 0–12 scale of vascular comorbidity severity.

#### 2.2.6. Demographics

 Self-reported demographic characteristics included sex, age, race (recoded as White or non-White), and years of education (treated as a proxy for socioeconomic status).

### 2.3. Analyses

Structural equation modeling (SEM) [31] was used to assess the direct longitudinal relationships between baseline religious factors (subjective religiousness, religious attendance, religious media use, and prayer frequency) and depression severity after three months, as well as their indirect effects through the mediating paths of social support, stress, and baseline depression severity. A theoretical path model was constructed and specified using a series of linear equations, with SAS 9.1 PROC CALIS. The initial model was examined and refined to produce an optimized path model that was both theoretically coherent and provided a strong fit with the data. All SEM analyses were conducted based on a partial correlation matrix which treated all other variables described above (sex, age, race, education, religious denomination, and vascular health) as covariates in all model equations.

#### 2.3.1. Theoretical Model

In the initial theoretical model (Figure 1), all religious factors were treated as exogenous variables. Social support and average stress were treated as independent outcomes of these religious factors. Baseline MADRS was treated as an outcome of social support, average stress, as well as all religious factors. Finally, 3-month MADRS was predicted by all model variables. Thus, religious factors were theorized to operate on final depression severity both directly and indirectly through paths of social support, average stress, and initial depression severity.

#### 2.3.2. Multiple Imputation of Missing Data

Initial screening for missing data indicated that 9.6% of participants would be excluded due to listwise deletion. Multiple imputation of missing values, using the Markov Chain Monte Carlo (MCMC) method, was performed on variables in the model except for 3-month MADRS with PROC MI in SAS 9.1. The MCMC method imputes for each missing data point a series of randomly selected values based on the observed joint distributions of all other variables included in the model [32, 33]. Consistent with multiple imputation theory [34], five independent imputations were run, and all SEM analyses were performed separately on each of these imputed datasets and parameter estimates aggregated using SAS 9.1 PROC MIANALYZE.

## 3. Results

To be included in the present analyses, participants were required to have MADRS assessments both at enrollment and after 3 months of treatment, as well as having been administered with the DDES contemporaneous with the baseline MADRS. Data imputation and all analyses were based on 386 participants meeting these criteria. Descriptive statistics for raw data prior to imputation are presented in Table 1. Bivariate correlations between model variables are summarized in Table 2.

**Figure 2:**
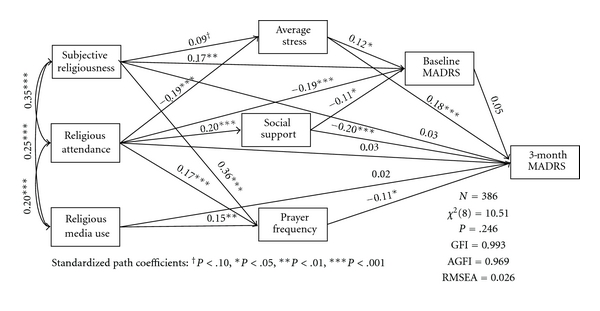
Path model with standardized coefficients for structural equation model of religious factors, social support, stress, and baseline and 3-month depression severity. MADRS: Montgomery-Åsberg Depression Rating Scale. Based on a partial correlation matrix controlling for gender, race, age, education, religious denomination, and vascular comorbidity. All variables except 3-month MADRS were measured at baseline. Error terms for endogenous variables are not reported.

SEM results for the initial theoretical model (Figure 1), *χ* ^2^(1) = 7.65, *P* = .006, GFI = 0.995, AGFI = 0.820, RMSEA = 0.128, indicated relatively poor model fit [31, 35, 36]. Modification of the model, guided by theory as well as by previous empirical findings, produced a more parsimonious model (Figure 2). Based on comparison of model fit indexes, several nonsignificant paths among mediators were removed. More significantly, the position of the prayer frequency in the model was altered. While model statistics showed that including prayer improved model fit, it was empirically unrelated to the psychosocial mediators specified in the model, in contrast with the other religious factors. Optimal fit was achieved by treating prayer as endogenous factor predicted by each of the other religious factors. While this approach differs slightly from the initial model, it is theoretically consistent with other research that treats prayer as a behavioral factor influenced by more general forms of religious engagement [37, 38]. Model statistics indicated very good fit for this optimized model, *χ* ^2^(8) = 10.55, *P* = .229, GFI = 0.993, AGFI = 0.972, RMSEA = 0.020.

## 4. Discussion

These results show a complex relationship between religion and severity of depression following treatment among older adults. While the results are generally in support of the hypothesis that stress and social support serve as psychosocial mediators of the relationship between religion and depression severity outcomes, they also indicate that religion has some unique effects not mediated by other variables in the current model. Furthermore, certain elements of religion appeared to have both harmful and beneficial effects on depression, via different mediating paths. 

Among the baseline religious factors in this model, only prayer frequency showed a significant direct relationship with depression severity after 3 months. Other religious factors (subjective religiousness, religious attendance, and religious media use) were found to be related to prayer frequency, average stress, and social support, which were in turn significantly related to 3-month depression severity, and thus appeared to be related only indirectly. While all of the significant effects of religious attendance, religious media use, and prayer frequency observed in this model showed inverse associations with depression severity, subjective religiousness appeared to be associated with worse depression through the paths of higher average stress and baseline depression severity, as well as with less severe depression through the path of higher prayer frequency. Overall, the final model supports the hypotheses that religion is longitudinally related to depression severity both directly and indirectly through its influence on other key psychosocial mediators, and that the net effect of these religious factors is beneficial after accounting for these direct and indirect pathways.

There were noteworthy differences between the associations of religious factors and depression severity before and after treatment. While subjective religiousness and religious attendance were both related directly to baseline depression severity, they were only indirectly and more weakly related to depression after treatment. By contrast, baseline prayer frequency was unrelated to baseline depression, but was significantly related to lower severity 3 months later. Religious media use showed no direct relationship to depression severity at either point, but was related to prayer frequency, which in turn was related to 3-month depression severity. Because the associations of religious factors with pre-treatment depression severity are cross-sectional, there is some ambiguity in their interpretation. It is possible that being in an acute state of depression had an influence on patients' religious practices, for example, by discouraging them from attending worship services, or increasing subjective religiousness. Another possible interpretation of these findings is that there are different specific facets of religiousness at work in influencing the severity of a depressive episode, distinct from those influencing response to treatment. Alternatively, it is possible that certain elements of religiousness influence the threshold of severity at which individuals decide to seek treatment. More research is needed to address these possible explanations.

Consistent with previous research [30, 39], lower reported stress and higher perceived social support at baseline were related to lower depression severity after 3 months, and these mediators appeared to partially account for the association of religious factors with depression severity. These results are consistent with the hypothesis that attending religious worship provides one with more opportunities to receive community support, and also helps to reduce stress. It is less clear why greater subjective religiousness appears to be associated with more stress and worse baseline depression, although this is consistent with findings from some other recent studies [16, 17]. One speculative interpretation is that individuals for whom religion is a highly central element of personal identity may be more prone to interpret their problems as signs of moral failure or divine punishment, particularly if they are also depressed, eliciting more intense feelings of stress than those who attribute their problems to more mundane causes.

It is especially noteworthy that the relationship between more frequent prayer and less severe depression after 3 months did not appear to be mediated by any stress-buffering effect, and that it also provided a pathway for indirect beneficial effects of subjective religiousness, religious attendance, and religious media use that were likewise independent of stress and social support. The mechanism by which prayer might ameliorate depression severity is not clear from this study. Recent research suggests that engaging in regular meditation may cause biological changes promoting better health in regions of the brain that are also related to depression [40, 41]. To the extent that prayer engages similar processes, this may suggest a potential biological mediator for this relationship. While there are physiological similarities in the practice of specific types of meditational [42] and ritual [43] prayer with meditative practice, the general category of prayer encompasses a wide range of other specific forms [44, 45] which were not differentiated by the measures used in this study, and thus further research is necessary to explore this hypothesis. Another possibility is that prayer helps people with depression to cope better with their illness and the factors that may have precipitated that illness. A natural next step suggested by these results is to examine more closely the specific types of prayer used by older adults in coping with depression, while at the same time measuring biomarkers known to influence treatment outcomes, to assess the hypothesis that certain prayer practices have physiological effects that are independent of the social support and cognitive coping facets of religion.

A further possibility is that prayer interacted in some way with the treatment that the patients were receiving for their depression, improving the effectiveness of that treatment. For example, it may have provided time for reflection on elements of therapy, or may have in some way promoted medication adherence. Since treatment details were not collected as part of this study, it is unfortunately not possible to examine these possibilities more closely with the present data. Regardless of the precise mechanism at work, the finding of a pattern of independent longitudinal effects on depression severity via multiple paths for each of the four religious factors measured in this study serves to highlight the complex and multifaceted nature of religion as a construct, and to reinforce the importance of taking multiple forms of religiousness into account when modeling its relationship with mental health. While the specific focus of this study is on late life depression, it is likely that these facets of religion operate in a similar way earlier in life. Nevertheless, the general pattern of increasing religiousness during older adulthood [37], alongside possible differences in the etiology of some forms of late life depression, compared with those developing earlier [46], make it important to empirically evaluate the applicability of this model in younger adults.

**Limitations**

Although depression severity outcomes were measured after 3 months of treatment, measurement of both the religious factors and the psychosocial mediators was conducted only at the time of the initial assessment. This leaves open questions of directionality among those model variables. For example, it is plausible that more perceived stress and worse initial depression contribute to increased subjective religiousness by prompting to patients to engage in more intense religious coping. A more comprehensive longitudinal study, ideally following individuals' religiousness, perceptions of stress, and social support prior to becoming depressed, would be needed to fully test these possibilities. Additionally, data regarding medication adherence and substance use were not available, making it impossible to test the possibility that religious factors influenced treatment outcomes by way of these potential mediators. Other limitations relate to the representativeness of the sample used in this study. Participants were receiving treatment at a major academic medical center, and may differ in important ways from depressed patients treated in other settings, or who never seek treatment. Furthermore, all patients were residents of the Southeastern US. This region is both more religious overall and more homogeneous in terms of religious group affiliation than other regions of the US [47], and thus the importance of religious factors in mental health outcomes seen here may not be fully generalizable to patients from other areas.

## 5. Conclusions

This study has both theoretical and methodological implications regarding research in religion and mental health. Theoretically, it provides evidence of a relationship with late life depression that is partially mediated through psychosocial pathways of stress buffering and social support, but also appears to have unique effects not explained by these factors. Methodologically, it demonstrates some of the complexity of the construct of religion, and its potentially multidirectional relationship with important outcomes. The present results contribute to a growing body of findings demonstrating that different facets of religiousness may have either harmful or beneficial relationships with mental health. Research that conceptualizes religiousness as a single construct, or that relies on a single facet such as worship service attendance, is thus likely to produce an incomplete picture of this relationship. In particular, this study suggests that subjective religiousness may be related to depression via multiple pathways in contrary directions, while private prayer appears to have a relationship that cannot be accounted for by any association with stress buffering. More research is needed to fully understand this network of relationships, as well as to identify other facets of religiousness that may impact mental health, and potential mechanisms to account for the residual relationships not explained by stress and social support.

## Figures and Tables

**Table 1:** Descriptive statistics.

	*N* ^ a^		
*Demographics*			
Female, *n* (%)	254	(65.8)	386
White, *n* (%)	329	(85.2)	386
Age (years), mean (SD)	69.40	(7.26)	386
Education (years), mean (SD)	13.65	(2.95)	386
Vascular comorbidity^b^, mean (SD)	1.46	(1.86)	384

*Depression*			
MADRS at baseline, mean (SD)	25.88	(7.84)	386
MADRS at 3 months, mean (SD)	12.57	(8.67)	386

*Mediators*			
Social support^c^, mean (SD)	22.99	(3.81)	364
Average stress^d^, mean (SD)	6.60	(2.08)	378

*Religious factors*			
Subjective religiousness^e^, mean (SD)	1.58	(0.67)	380
Religious attendance^f^, mean (SD)	2.67	(1.78)	380
Religious media use^f^, mean (SD)	1.68	(1.93)	379
Prayer frequency^f^, mean (SD)	3.08	(1.81)	378
Mainline Protestant, *n* (%)	152	(40.4)	376
Conservative Protestant, *n* (%)	129	(34.3)	376
Catholic, *n* (%)	36	(9.6)	376
Other religious affiliation, *n* (%)	33	(8.8)	376
No religious affiliation, *n* (%)	26	(6.9)	376

^a^ *N* with nonmissing data prior to imputation.

^b^Scored on a 0–12 scale.

^c^Scored on a 10–30 scale.

^d^Scored on a 1–10 scale.

^e^Scored on a 0–2 scale.

^f^Scored on a 0–5 scale.

**Table 2:** Bivariate correlations between study variables.

	(2)	(3)	(4)	(5)	(6)	(7)	(8)
(1) subjective religiousness	0.47***	0.41***	0.60***	0.04	0.08	0.13**	0.03
(2) religious attendance		0.29***	0.43***	−0.14**	0.21***	−0.15**	−0.06
(3) religious media use			0.41***	0.06	−0.02	0.06	0.05
(4) prayer frequency				−0.002	0.11*	0.06	−0.05
(5) average stress					−0.19***	0.18***	0.23***
(6) social support						−0.15**	−0.25***
(7) Baseline MADRS							0.13**
(8) 3-Month MADRS							
